# Enhancing photon generation rate with broadband room-temperature quantum memory

**DOI:** 10.1038/s41598-022-25060-1

**Published:** 2022-12-19

**Authors:** Chao-Ni Zhang, Xiao-Ling Pang, Jian-Peng Dou, Hang Li, Tian-Huai Yang, Xian-Min Jin

**Affiliations:** 1 Center for Integrated Quantum Information Technologies (IQIT), School of Physics and Astronomy and State Key Laboratory of Advanced Optical Communication Systems and Networks, Shanghai Jiao Tong University, Shanghai, 200240 China; 2 Hefei National Laboratory, Hefei, 230088 China; 3 TuringQ Co., Ltd., Shanghai, 200240 China; 4 Chip Hub for Integrated Photonics Xplore (CHIPX), Shanghai Jiao Tong University, Wuxi, 214000 China

**Keywords:** Quantum information, Single photons and quantum effects

## Abstract

Photons with high generation rate is one of the essential resources for quantum communication, quantum computing and quantum metrology. Due to the naturally memory-built-in feature, the memory-based photon source is a promising route towards large-scale quantum information processing. However, such photon sources are mostly implemented in extremely low-temperature ensembles or isolated systems, limiting its physical scalability. Here we realize a single-photon source based on a far off-resonance Duan-Lukin-Cirac-Zoller quantum memory at broadband and room-temperature regime. By harnessing high-speed feedback control and repeat-until-success write process, the photon generation rate obtains considerable enhancement up to tenfold. Such a memory-enhanced single-photon source, based on the broadband room-temperature quantum memory, suggests a promising way for establishing large-scale quantum memory-enabled network at ambient condition.

## Introduction

High generation rate of photon source is highly related to the accessible scale of quantum systems and plays a key role in advances stepping to large-scale quantum information processing, enabling quantum communication^1–3^, quantum computing^4–6^, and quantum metrology^7,8^ beyond the proof-of-principle demonstrations. Up to date, photon sources have been realized in many different systems^9–13^ and numerous schemes are proposed to improve their performance to approach the ideal one^14–16^. Despite great progresses in the development of photon source, scaling up the quantum information processing remains exponentially hard due to the transmission loss in channel and probabilistic nature of quantum operation. A promising solution for this scalability problem is to cooperate single-photon sources with quantum memory, where photons can be stored and retimed in a controllable fashion to gate the probabilistic nature.

This quantum memory-enabled strategy was widely applied in kinds of quantum information tasks, like boosting synchronous multiphoton rate from nondeterministic sources^17,18^. In these cases, each photon source is managed to interface with an external quantum memory, while high performance of both elements as well as their compatibility should be simultaneously satisfied to obtain high-fidelity and efficient operation. The memory-based single-photon source^12,19,20^, whose dynamics directly begins with the creation of stationary excitation in particle and later conversion to optical quanta, is considered to be a more integrated version. The combination of the single-photon generation and storage is free from the requirement of preparing external source as well as the strict matching of optical and matter mode, therefore more straightforward and promising to achieve large scale.

In the past decades, the development of the memory-based single-photon source has laid emphasis on suppressing decoherence and noise during the generation process for high performance, motivating numerous progresses on ultra-low temperature and well-isolated systems^19–22^. However, the complexity and size of the physical systems grow quickly with the number of controlled quantum units, which may limit its further application in large scale quantum networks. Operation simplicity and better scalability can be obtained with room-temperature systems, but the loss and decoherence induced by atomic thermal motion impedes long lifetime and high retrieval efficiency of stored collective excitation^23^, and meanwhile the noise originating from the fluorescence and atomic collision^24^ deteriorates the fidelity of the obtained photons to an unacceptable level. Therefore, how to well preserve the collective excitation in presence of severe decoherence and extract signal photons from strong noise has been a long-standing challenge for making room-temperature memory-based photon source work in quantum regime. Recently, efforts have been made in implementing room-temperature single-photon sources with built-in memory^25^, and a further enhancement of photon generation, especially when the broadband feature allows operations at a high-data rate, will make it more appealing for real-life applications.

Here, with a far off-resonance Duan-Lukin-Cirac-Zoller (FORD) configuration^24^, we experimentally demonstrate the enhancement of photon generation rate with broadband room-temperature quantum memory. The memory-built-in photon source proceeds repeat-until-success write process via high-speed feedback control. The success rate of stored excitation associated with photon emission is boosted up to ten times higher, meanwhile the nonclassical character of photons can be well retained.

## Results

**Figure 1:**
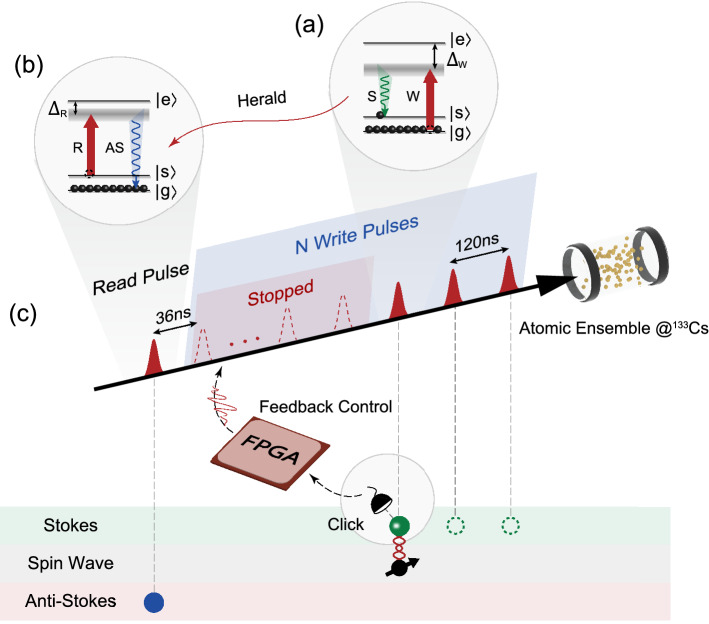
The schematic diagram of enhancing photon generation rate with broadband room-temperature quantum memory. (**a**) The write process. In the three-level $\Lambda$-type configuration, the ground state $\left| g\right\rangle$ and $\left| s\right\rangle$ respectively represent $F=3$ and $F=4$ hyperfine state of $6S_{1/2}$ state, while the manifold of $6P_{3/2}$ state is labeled as $\left| e\right\rangle$ state. The gray zone represents the broad virtual state addressed by the strong write pulse, and the emitted Stokes photon is painted in green. (**b**) The read process. The generated anti-Stokes photon is painted in blue. (**c**) The main idea of feedback protocol is to extend the single write pulses into a sequence of write pulse, and it keeps inducing Stokes photons until one is successfully detected, leading to a higher efficiency of creating the spin wave.

The generation process of the heralded single photon is schematically illustrated in the Fig. 1. Initially all atoms are prepared in the ground state $\left| g\right\rangle$ by a pump light. Then an off-resonant write pulse is incident on the atomic ensemble, and it aims at inducing a Stokes photon via spontaneous Raman scattering (SRS), heralding the creation of a correlated collective excitation among the atoms. In this SRS process, it is worth noting that the joint state of the generated atom-photon pair is of the two-mode squeezer type, which can be described as^26,27^

1 $$\begin{aligned} \left| \Psi _{\text{ joint } }\right\rangle =\sqrt{1-p_0}\left( \left| 0_p\right\rangle \left| 0_a\right\rangle +\sqrt{p_0}\left| 1_p\right\rangle \left| 1_a\right\rangle +p_0\left| 2_p\right\rangle \left| 2_a\right\rangle +o\left( p_0^{3 / 2}\right) \right) \end{aligned}$$

where $p_0$ is the excitation probability of generating Stokes photons in the write process. $\left| n_p\right\rangle$ denotes *n* Stokes photons generated while $\left| n_a\right\rangle$ refers to *n* atomic excitations among the ensemble. Due to the photon number non-resolving detector applied in the Stokes channel, it is impossible to distinguish the multiphoton state from the single-photon state. Along with loss and inefficient detection of the Stokes photons, the high-order excitations will be falsely recognized as the desired collective excitation, resulting in multiphoton component of the single-photon source. To suppress the high-order excitations, we adopt a weak write pulse to obtain low excitation probability, which is about $1.3 \%$ in this experiment. In this case, the probability of high-order excitations events ($\sim p_0^2$) is negligible comparing with that of single excitation event ($\sim p_0$). The generated atomic state now can be described as^19,24^

2 $$\begin{aligned} \left| \Psi \right\rangle \mathrm{{ = }}\frac{1}{\sqrt{N}} \sum _{j=1}^{N} \mathrm {e}^{\mathrm {i}\left( \vec {k}_{\mathrm {W}}-\vec {k}_{\mathrm {S}}\right) \cdot {\vec r}_j}\left| g_{1} g_{2} \cdots s_{j} \cdots g_{N-1} g_{N}\right\rangle \end{aligned}$$

where $\vec {k}_{\mathrm {W}}$ and $\vec {k}_{\mathrm {S}}$ respectively denotes the wave vectors of the write pulse and Stokes photon, and ${\vec r}_j$ is the position vector of the excited atom. After a programmable storage time, a read pulse is applied to convert the collective excitation into a flying anti-Stokes photon. Experimental details are in the Methods.

To enhance the generation rate of the anti-Stokes photons, we incorporate the repeat-until-success write process into the FORD quantum memory. As shown in Fig. 1c, instead of making a single write attempt in each period, a string of *M* pulses is programmed to be incident into the atomic ensemble. When the former write pulse fails to induce a SRS process, the latter write pulse is applied to make another attempt. The cycle ends until a Stokes photon from a successful write process is detected, triggering the feedback control to stop the rest of the write pulses (see Methods for more details). The generated collective excitation is stored in the memory, and a read pulse at fixed moment is applied to retrieve the anti-Stokes photon. In this case, the probability of creating a collective excitation in all trials is much higher than the probability with one write pulse ($P_{\mathrm {S}}$), approaching an enhancement rate up to $\sum \limits _{k = 1}^M {\left( 1-{P_{\mathrm {S}}} \right) }^{k-1}$.

**Figure 2:**
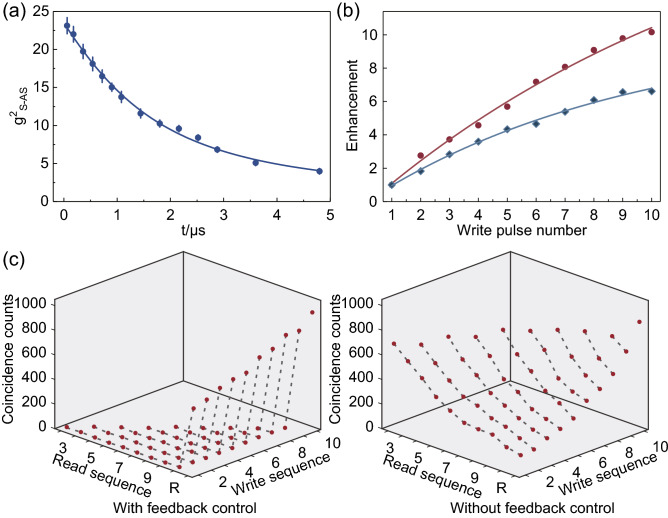
Performance of the FORD memory and measured enhancement with or without feedback control. (**a**) The cross-correlation measurement. The depicted data are obtained under the condition of one write pulse and one read pulse. The cross-correlation function $g_{\mathrm {S}-\mathrm {AS}}^{(2)}$ decreases due to the decoherence arising from the atomic motion. And the data are fitted by the function $g_{\mathrm {S}-\mathrm {AS}}^{(2)}=1+C/\left( 1+\mathrm {At}^{2}+\mathrm {Bt}\right)$, in which the quadratic term originates from the random atomic motion and the linear term results from the background noise of incident pulses. Error bars are derived from the Poisson distribution of photons. (**b**) The enhancement of generation rate as a function of the number of write pulses. Here the data in red dots correspond to the results with feedback control, while the data in the blue diamonds are obtained from the experiments without feedback control. The enhancement is defined as $E={R_{k}}/{R_{1}}$, where the $R_{1}$, $R_{k}$ represents the photon generation rate in the experiment with one and *k* write pulses respectively. (**c**) The possible coincidence counts between different pulses in two experiments. The data in both cases are obtained with an efficient measurement period of 300 s, which corresponds to $10^{8}$ experiments preformed. In the horizontal axis labeled by write (read) sequence, the number *n* represents the nth write pulse, and in the read sequence R means the applied read pulse. The measured counts of anti-Stokes photons, which are converted from the collective excitation generated by the same order write pulse, is connected with a dashed line.

As the memory is the central elements in the whole generation process, its performance, especially the noise level and the lifetime, determines the single-photon purity and the accessible enhanced generation rate. We measure the second order cross-correlation function $g_{\mathrm {S}-\mathrm {AS}}^{(2)}$^28^ between Stoke photons and anti-Stokes photons to evaluate the storage performance of the FORD memory, shown in Fig. 2a. The lifetime, defined as the time interval for the cross-correlation dropping to 1/*e* of the initial value^29–31^, sets an upper limit for the time span of incident pulse sequence, and the measured lifetime is 2.3$\mu s$. Owing to the broadband feature of the FORD memory and high speed feedback control, a short write pulse is used and the time interval between two adjacent write pulses is reduced to 120 ns. Therefore, dozens of write processes can be accommodated within the lifetime for high enhancement. Moreover, the cross-correlation $g_{\mathrm {S}-\mathrm {AS}}^{(2)}$ are all over 8 within the lifetime, much larger than the classical boundary 2, being able to violate the Cauchy-Schwarz inequality^32^. This high cross-correlation indicates a low noise level^12,21^.

Having characterized the performance of the quantum memory, we perform the experiments with write pulse number ranging from 1 to 10. In the ideal condition of constant retrieval efficiency, the enhancement of the photon generation rate approximately linearly increases with the number of write pulses when $P_{\mathrm {S}}$ is small. As the red dots shown in the Fig. 2b, the rising tendency of measured enhancement is close to the linear trend. It indicates that, though the average retrieval efficiency decreases with the number of write pulses, the enhancement of the probability of generating collective excitation now is the dominant factor to determine the generation rate, and further extension of write pulse sequence can still help to effectively access a higher generation rate. To investigate the role of the feedback control in the enhancement of the generation rate, we also measure the enhancement value without feedback control for comparison, as shown by the blue diamonds in Fig. 2b. In this case, the pre-programmed pulse sequence is same as the one applied in the feedback protocol. The only difference is that the feedback control is removed, allowing all write pulses interacting with the atomic ensemble one by one. As shown in Fig. 2b, the measured enhancement without feedback control is found always smaller than that with the feedback control, and the difference becomes larger as the write pulses number increases.

For further characterizing the difference with or without feedback control, we choose the experiments with 10 write pulses to reveal the detrimental effect of enhancement. All possible coincidence counts $N_{\mathrm {S}_{i}-\mathrm {AS}_{j}}$ between different pulses are shown in the Fig. 2c, where $N_{\mathrm {S}_{i}-\mathrm {AS}_{j}}$ means the counts of anti-Stokes photon retrieved by the pulse *j*, conditioned on the collective excitation generated by the write pulse *i*. For the experiment with feedback control, the coincidence counts between different write pulses are close to zero while the coincidence counts between write pulse and read pulse are considerable, indicating the generated collective excitation is exclusively retrieved by the read pulse. However, for the experiment without feedback, the results show a different trend. The coincidence counts between different write pulses are considerable, and the coincidence counts between write pulse and read pulse here are smaller than those in the former condition. It means that, in this case, the subsequent write pulses read the stored collective excitation out and thus less anti-Stokes photons are obtained when the read pulse genuinely works.

**Figure 3:**
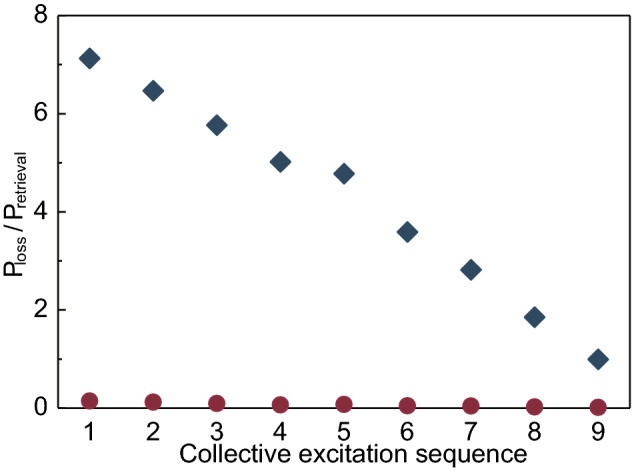
The details of the loss caused by the write pulses. In the horizontal axis labeled by collective excitation sequence, the number n represents the collective excitation generated by the nth write pulse. The results from experiment with feedback control is represented in red dots and the blue diamonds refer to the results from the experiment without feedback control. In principle, the results from the feedback protocol are zero, and the small violation here mainly comes from the environmental noise and the dark counts of the detector.

For each created collective excitation, we sum up the retrieval efficiency of all subsequent write pulses ($P_{\text{ loss }}$) and calculate the ratio with the retrieval efficiency of the read pulse in feedback protocol ($P_{\text{ retrieval }}$). As shown in the Fig. 3, the ratio is up to 7.1 times for the first collective excitation and remains almost commensurate for the ninth collective excitation, implying the importance of removing the rest write pulses to protect the excitation.

**Figure 4:**
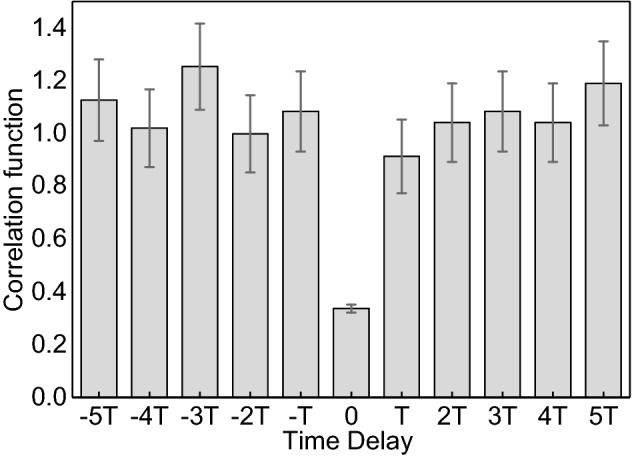
Measured second-order correlation function of enhanced single-photon source. We choose the experiment data with 10 write pulses, and error bars represent one standard deviation assuming Poisson distribution of photons.

Besides the generation rate, the purity of the heralded single photon is important for its applications in quantum information processing^33–37^. We quantify the single-photon character by measuring the anti-correlation function with the Hanbury Brown-Twiss (HBT) interferometer^38,39^, as shown in Fig. 4. The measured result is $0.336 \pm 0.015$ with 10 write pulse sequence, showing that in most case there is only one heralded anti-Stokes generated. Moreover, thanks to the time-of-flight data recording module (see Methods for more details), we can obtain the correlation between anti-Stokes photons generated in different trials. The second-correlation function between different trials is around 1, indicating that the generation processes are mutually independent events.

## Discussion

In summary, we present an experimental demonstration of enhancing photon generation rate with broadband room-temperature quantum memory. We successfully observe about tenfold enhancement by introducing the repeat-until-success write process via high-speed feedback. We also test the nonclassical character of the generated single photons, and the result shows that the non-classicality is retained while the generation rate rises considerably. Enhancing photon generation rate by repeat-until-success protocol, the lifetime of the quantum memory is quite important for the achievable photon rate and the quality of the generated photons. For room-temperature systems, the decoherence caused by the atomic thermal motion is the main barrier for obtaining long lifetime^40^. By reducing the loss originating from the motional atoms leaving the interaction region, the utilization of larger beam waist of control pulses or a small-diameter cell^23^ has allowed to prolong the lifetime. Collisions between atoms and container walls is harmful to the atomic polarization, and an effective approach is anti-relaxation coating of the vapor cell walls to preserve the atomic polarization^41,42^. In addition, a recent progress reports development toward transferring the collective excitation of electronic spins to the long-lived noble-gas nuclear spins via spin exchange, promising the lifetime extension to hour-long regime^43^. Besides the primary purpose of generating single photons, our system, along with the memory built-in feature, can also serve as a building block for preparing synchronized multi-photon state and room-temperature quantum repeater, making it an versatile elements for further applications in future quantum network.

## Methods

Experimental details The 75-mm-long cell containing billions of cesium atoms is placed in a three-layer magnetic shielding to minimize the magnetic field. By adjusting the current of heater wrapped around the cell, it is maintained at the temperature of 61.3 $^{\circ }$C to get a large optical depth of 5000. In our system, the repeat-until-success protocol needs quickly varying the write pulse sequence, and thus we utilize a high-speed eletro-optical modulator to generate write and read pulses. But the maximum input power and insertion loss of the eletro-optical modulator limits the achievable energy of the output write (read) pulses. After being fed in to a homemade tapered amplifier (TA), the energy of the write (read) pulses is boosted by 17 dB. Pump pulses come from another external cavity diode laser and is 1*us* generated by an acousto-optical modulators (AOM). The pump pulse is resonant with the transition $6 S_{1 / 2}, F=4 \rightarrow 6 P_{3 / 2}, F^{\prime }=4$ co 5 crossover of cesium, while the write (read) pulse is red detuned $4 \mathrm {GHz}$ from this transition. Due to their orthogonal polarization^44^, we can preliminary separate the Stokes and anti-Stokes photons from strong write (read) light via a Wollaston prism. To obtain signal photons with high fidelity, further purification by cascaded Fabry-Pérot cavities is needed. There are three Fabry-Perot filters in one cascade-cavity filter in our experiment. The measured peak transmissivity of single Fabry-Perot filter is about $90\%$, and the overall transmission efficiency of the cascaded-cavity filter is about 70%. In addition, the whole signal noise ratio of the cavity system is up to $10^{7}:1$. We use silicon avalanche photodiodes from Excelitas Technologies to detect signal photons, and the quantum efficiency is about 50% at 852 nm.

As mentioned before, a long lifetime of quantum memory is the vital parameter to achieve high photon generation rate in this repeat-until-success protocol. Here the lifetime is mainly restricted by the atomic motion since the excited atom rapidly leaves the interaction region. We add 10 Torr Ne buffer gas into the cell to slow down the diffusion of cesium atoms and more importantly adopt a larger write (read) pulse to create a larger interaction region, prolong the lifetime from 800 ns to 2.3 $\mu s$. Note that a longer lifetime is accessible if we further increase the beam waist of write (read) pulse. But it will result in a lower retrieval efficiency as the energy density of the pulse decreases. The trade-off can be solved by applying a laser with higher power. Based on present configurations, we set the beam waist of write (read) pulse of 393 $\mu m$ for obtaining both acceptable lifetime and retrieval efficiency.

*Feedback control* The generation system of write pulse utilizes a high-speed eletro-optical modulator to chop the continuous laser into any desired pulse sequence, which is driven by the external electrical signal. When applying an electrical signal, the applied electrical signal induces phase change in both arms of this Mach-Zehnder eletro-optical modulator, which in turn results in the generation of an optical pulse at the output of the modulator through constructive interference. Otherwise, the phase difference between two arms of the modulator leads to destructive interference and thus no control light is output. To implement the repeat-until-success protocol, the output of the detector in Stokes channel in sent into the Field Programmable Gate Array (FPGA) and meanwhile the drive signal of eletro-optical modulator is from the FPGA. Assuming at the beginning of a new period, the FPGA sends Digital electrical signal to the eletro-optical modulator to generate the write pulse at set time. The generated write pulse is guided to the atomic ensemble for creating the collective excitation. If it succeeds and meanwhile the emitted Stokes photon is detected by the single photon detector. The output of the detector in Stokes channel is first sent to a voltage amplitude discriminator, mapping the irregular electrical signal into a digital one, and then the generated digital signal is sent into the FPGA. According to the setted program inside, the FPGA will not generate electrical signal to drive eletro-optical modulator generate another write pulse. Otherwise, the whole system repeats the above process again. In our experiment, the time interval between adjacent write pulses is mainly determined by the optical path of Stokes photon, the response time of the detector and the transmission time of the electric signal. Here we set this interval (120 ns) a bit longer than the sum of overall items in order to stop the remaining pulses in time.

*Time-of-flight data recording module* In our experiment, the electric signal of detected Stokes and anti-Stokes photons is transmitted to the time-of-flight data recording module, where the channel and time message of every registering photon is recorded in a constant format and then stored in the connected computers. Due to the high time resolution (64 ps) of this instrument, the photon flow can be faithfully reproduced from the kept data, and any further analysis, including counting the coincidence counts between the Stokes and anti-Stokes photons within the given time windows and getting the temporal distribution of specified photon signal, can be easily realized by relative data processing program. Moreover, owing to the high-speed data recording ability of this recording module, the data processing program can operate while measuring the data to get a real-time results.

## Data Availability

The data that support the findings of this study are available from the corresponding author on request.
